# Supplementary material for: Assessment of the Performance of Lactate Dehydrogenase-Based Rapid Diagnostic Test for Malaria in Djibouti in 2022–2023
Source: Diagnostics (Basel). 2024 Jan 25;14(3):262. doi: 10.3390/diagnostics14030262 (PMC10854848; doi:10.3390/diagnostics14030262)
Supplement: Supplementary file 1 [file diagnostics-14-00262-s001.zip › diagnostics-2738141-supplementary.pdf]

Table S1: Prevalence of *P. falciparum* and *P. vivax* from RDT-LDH based diagnosis and QPCR diagnosis in Djibouti city (2022-2023).

	**2022**	**2023**
Plasmodium falciparum		
RDT_PF+	40	67
TOT	198	153
%	20.2%	43.8%
QPCR_PF+	53	76
TOT	198	152
%	26.8%	50%
Plasmodium vivax		
RDT_PV+	14	13
TOT	198	153
%	7.1%	8.5%
QPCR_PV+	14	15
TOT	198	152
%	7.1%	9.9%

RDT_PF+: number of positive diagnoses of *Plasmodium falciparum* by Rapid Diagnostic Test. QPCR_PF+: number of positive diagnoses of *Plasmodium falciparum* by Quantitative Polymerase Chain Reaction. TOT: total number of samples tested used as the denominator to calculate the percentage (%). RDT_PV+: number of positive diagnoses of *Plasmodium vivax* by Rapid Diagnostic Test. QPCR_PV+: number of positive diagnoses of *Plasmodium vivax* by Quantitative Polymerase Chain Reaction.
